# Mass of Abrikosov vortex in high-temperature superconductor YBa$_2$Cu$_3$O$_{7-\delta }$

**DOI:** 10.1038/s41598-021-00846-x

**Published:** 2021-11-05

**Authors:** Roman Tesař, Michal Šindler, Christelle Kadlec, Pavel Lipavský, Ladislav Skrbek, Jan Koláček

**Affiliations:** 1 Institute of Physics, Czech Academy of Sciences, Na Slovance 2, 182 21 Prague 8, Czech Republic; 2 Faculty of Mathematics and Physics, Charles University, Ke Karlovu 3, 121 16 Prague 2, Czech Republic

**Keywords:** Superconducting properties and materials, Superconducting properties and materials, Terahertz optics

## Abstract

For more than four decades, mass of Abrikosov vortices defied experimental observations. We demonstrate a method of its detection in high-temperature superconductors. Similarly to electrons, fluxons circulate in the direction given by the magnetic field, causing circular dichroism. We report the magneto-transmittance of a nearly optimally doped thin YBa$_2$Cu$_3$O$_{7-\delta }$ film, measured using circularly polarized submillimeter waves. The circular dichroism emerges in the superconducting state and increases with dropping temperature. Our results confirm the dominant role of quasiparticle states in the vortex core and yield the diagonal fluxon mass of $2.2 \times 10^8$ electron masses per centimeter at 45 K and zero-frequency limit, and even larger off-diagonal mass of $4.9 \times 10^8 m_e$/cm.

The limitations of today’s digital technology will likely be overcome by ultra-fast superconductive electronics^1^, quantum circuits^2^, and quantum information processing. Such advanced electronic devices can operate within the framework of *fluxonics* taking advantage of intrinsic properties of superconducting vortices. Abrikosov vortices, or fluxons, are ideal information carriers: they are topologically stable and keep a uniform nano-scale size given by quantum conditions^3,4^.

Nevertheless, the fluxon mass is a particularly controversial issue. Its theoretical estimates are scattered over more than eight orders of magnitude. Vortex mass in superconductors has been debated for over 50 years since its theoretical prediction by Suhl^5^, $4000~m_e/$cm for Nb, where $m_e$ is the electron mass. Multiple theories emerged in 1991, stimulated by the discovery of high-$T_c$ superconductors; for instance, the vortex mass in YBaCuO was predicted^6^ to reach $10^8\,m_e/$cm. Rather different trends follow from the theory of Han et al.^7^, which gives $10^{13}\,m_e$/cm in Nb at 5 K, while for YBaCuO at the same temperature, it yields $3\times 10^9\,m_e$/cm. Additionally, the vortex mass can be increased by backflow effects^8^ or by the strain field^9,10^ with estimates for YBaCuO from dominant $10^{10}$ to negligible $10^4\,m_e/$cm. We also note that theories for the mass of quantized vortices in closely related superfluids offer similarly conflicting results^11,12^ that span the full range from the infinite mass^13–15^ to the negligible one^16^.

In contrast to the plethora of theoretical predictions of the effective vortex mass in type-II superconductors, we are aware of only two relevant experiments, both supporting rather large values. Fil et al.^17^ measured the acousto-electric effect in YB$_6$ and deduced a vortex mass of $10^{10}\,m_e/$cm. Golubchik et al.^18^ observed the movement of individual vortices in a superconducting Nb film near its critical temperature by combining high-resolution magneto-optical imaging with ultrafast heating and cooling. A nonzero mass is indispensable to explain their data; they estimated the fluxon mass to be in the interval $(0.3 - 6) \times 10^8~m_e$/cm.

The extremely large spread in theoretically predicted values and scarce experimental data available motivated us to independently determine the mass of a fluxon. Here, we present relevant experiments leading to the evaluation of the vortex mass in a nearly optimally doped YBa$_2$Cu$_3$O$_{7-\delta }$. Our approach is inspired by the cyclotron resonance measurement, a method commonly employed to obtain the effective mass of charge carriers in semiconductors. Exposed to an external magnetic field, charged particles move in circular orbits and, under certain conditions, resonantly absorb monochromatic radiation. In the case of fluxons, however, the cyclotron motion is induced by interaction with circularly polarized far-infrared laser light. This interaction depends on the sense of circular polarization and manifests itself as a differential transmittance for clockwise and anti-clockwise polarized waves, known as magnetic circular dichroism. We show that the theory of Kopnin and coworkers reproduces our experimental data *without any fitting parameter*. Thus, our results support the theory of the fluxon mass developed by Kopnin and Vinokur^19^ and that of the Magnus force reduction by the factor of Kopnin and Kravtsov^20,21^.

## Far-infrared measurements of the fluxon mass

### Magneto-transmittance of circularly polarized light

**Figure 1:**
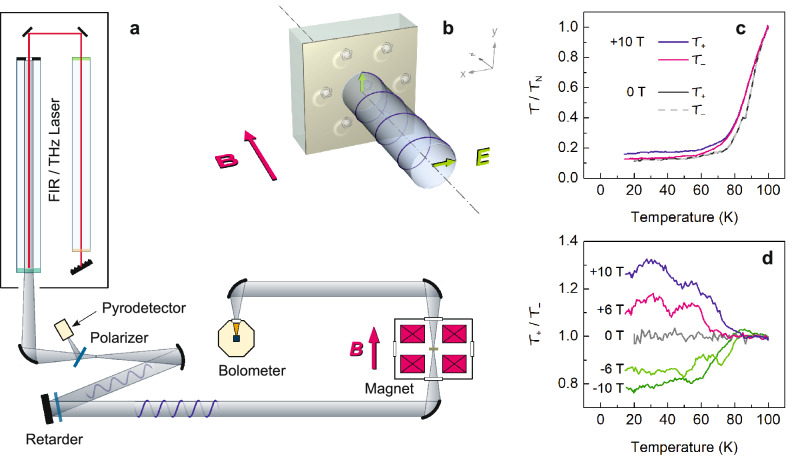
Far-infrared measurement of fluxon mass. (**a**) Sketch of the magneto-optical setup. The continuous laser beam is split by a linear wire-grid polarizer; the reflected part is monitored using a pyroelectric detector to keep trace of unavoidable power fluctuations, whereas the transmitted part proceeds toward the sample. The retarder converts the light from linear to circular polarization. The propagation of the circularly polarized beam and the magnetic field are perpendicular to the film surface, as detailed in panel (**b**). (**b**) Vortices in the film (gray circles) control the transmittance of the sample via the following mechanism: The electric field of the laser light drives the supercurrent. The Magnus force accelerates vortices in the direction perpendicular to the supercurrent; in reaction, the vortex motion affects the supercurrent and, thus, the transmittance. In the sketch, the electric field in the sample, as well as the vortices, rotate clockwise. If the light frequency is close to the cyclotron frequency of vortices, the motion of vortices is resonantly enhanced, leading to the observed dichroism. The extent of the cyclotron motion is strongly exaggerated; in fact, the fluxon circulates on a radius of less than $10^{-12}$ m at the strongest laser line. (**c**) Transmittance of the YBa$_2$Cu$_3$O$_{7-\delta }$ superconducting sample, normalized to the normal-state transmittance ${{\mathcal {T}}}_N$ at 100 K and plotted for two circular polarizations versus temperature. The dichroism is clearly visible below 70 K in a magnetic field of 10 T; in a zero field, no dichroism appears. (**d**) Transmittance ratio $\mathcal{T}_+/{{\mathcal {T}}}_-$ measured in several applied magnetic fields plotted versus temperature. The data were obtained using a 312 $\mu$m laser line ($6.1\times 10^{12}$ rad/s). Above the critical temperature, the dichroism is absent, showing that the normal-state Hall component is negligible.

We designed and developed a unique far-infrared (FIR) transmission experiment capable of probing the circular dichroism (see Fig. 1). The breakthrough that has eventually allowed us to conduct this research is a custom-made retarder^22^ inserted in the optical path near the FIR-laser output aperture; it delays the horizontal polarization relative to the vertical one by an adjustable phase difference. A mutual phase shift of $\pm \pi /2$ converts the THz beam of equal vertical and horizontal polarization components into the circularly polarized state. Since the phase delay introduced by the retarder is inversely proportional to the wavelength of the incoming light, each laser line requires a separate adjustment.

We measured the transmittance of the sample for several laser lines at wavelengths 119, 163, 312, 419, and 433 $\mu$m^23^, corresponding to the terahertz circular frequencies $\omega$ of 15.9, 11.6, 6.05, 4.50, and 4.35 $\times 10^{12}$ rad/s, respectively. Our setup shown in Fig. 1a enables fast flips between clockwise ($+$) and anti-clockwise (−) circular polarizations; consequently, we probed the transmittance $\mathcal{T}_+$ and ${{\mathcal {T}}}_-$ of both polarizations under identical conditions. The transmittance, i.e., the fraction of laser energy transmitted through the sample, is evaluated as the bolometer-to-pyrodetector signal ratio, effectively eliminating any possible time instability in the laser power. Identical profiles of both signals confirm that the transmittance is measured in the linear regime.

Our experimental protocol is as follows: In experimental runs, we apply a magnetic field at a temperature well above $T_c$. As the temperature drops, vortices freeze into a regular Abrikosov lattice. The applied magnetic field *B* is kept constant since any change in *B* would result in inhomogeneous patterns of the vortex density. The temperature *T* is swept down and up at a steady sweep rate of 2.5 K/min, the instantaneous temperature of the sample being recorded together with the transmittance. Care is taken to avoid hysteresis of the down and up sweeps.

Figure 1c displays a typical temperature behavior of the transmittance observed in the external magnetic field of 10 T using a 312 $\mu$m laser line. The results obtained with different laser lines are similar. As expected, the transmittance measured in zero field does not show any dichroism, in absence of Abrikosov vortices to induce an asymmetry. In nonzero fields, however, the low-temperature circular dichroism is clearly observed and can be attributed to the formation of vortices threading the sample. The effect is enhanced in higher applied magnetic fields, thanks to the growing areal density of Abrikosov vortices (see Fig. 1d). We focus on the low-temperature region, $T<50$ K, where the thermal quasiparticles play a negligible role and the response of the system is fully governed by the motion of fluxons.

### Sample specification by time-domain terahertz spectroscopy

**Figure 2:**
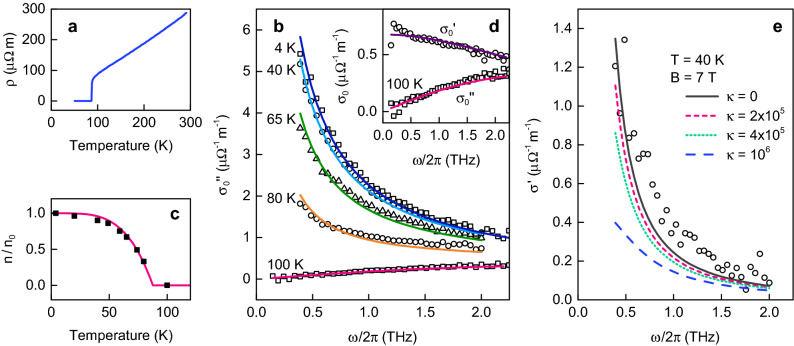
Sample specification. Panels (**a**–**d**) refer to the zero magnetic field. (**a**) The dc resistivity as a function of temperature provides the critical temperature $T_c=87.6$ K. Extrapolation of the normal-state resistivity below $T_c$ gives negligible residual resistivity. (**b**) The imaginary part of conductivity as a function of frequency for several temperatures. The two-fluid model (lines) reproduces the data (symbols) for the superconducting fraction $f_s=1-T^4/T_c^4$ (line), which is compared in (**c**) with the fitted values. (**d**) The real (circles) and imaginary (squares) components of the complex conductivity at 100 K provide the relaxation time $\tau _N=47$ fs. (**e**) The measured real part of conductivity for linearly polarized light at magnetic field 7 T (circles) compared with fits based on the vortex dynamics, $\sigma =\tfrac{1}{2}(\sigma _++\sigma _-)$, supports small pinning with $\kappa =2\times 10^5$ N/m$^2$.

We chose the most common high-$T_c$ material YBa$_2$Cu$_3$O$_{7-\delta }$ in the form of a thin film with a thickness of $L = 107$ nm, and with CuO$_2$ planes parallel to the surface. The sample was prepared at National Chiao Tung University (Taiwan) using a pulsed laser deposition method from a stoichiometric target on a lanthanum aluminate substrate oriented in the (100) plane. The substrate dimensions are $10 \times 10 \times 0.5$ mm$^3$. Several measurements were performed to establish sample properties. Figure 2 summarizes some relevant results.

The critical temperature of the film, $T_c = 87.6$ K, was determined from a dedicated measurement of dc resistivity $\rho$ (Fig. 2a). For our slightly underdoped sample, this $T_c$ corresponds to the hole density^24^ $n_0=1.68\times 10^{27}$/m$^3$. The linear slope of $\rho$ extrapolated to zero temperature shows negligible residual resistivity, so the relaxation time is inversely proportional to temperature.

Additional film properties were established in a separate experiment using standard time-domain THz spectroscopy. Broadband linearly polarized THz pulses (0.3–2.5 THz) were generated by exciting an interdigitated LT-GaAs emitter with a Ti:sapphire femtosecond laser beam at 800 nm^.25^ We measured the complex conductivity $\sigma =\sigma '+i\sigma ''$ for frequencies $\omega /2\pi$ in the range 0.5–2 THz at temperatures from 4 to 100 K (Fig. 2b,d,e). At the zero magnetic field and below $T_c$, the two-fluid model fit confirms the dominant London contribution, $\sigma _0\approx in e^2/(m\omega )$, with a temperature-dependent condensate density $n = n_0\,(1 - T^4/T_c^4)$, the hole mass $m=3.3~m_e$, and the elementary charge *e*. Comparing the conductivities at temperatures of 4 K and $T_N=100$ K in Fig. 2d, we found the relaxation time $\tau _N=5\times 10^{-14}$ s. Conductivities at all temperatures from 4 to 100 K are consistent with $\tau = \tau _NT_N/T$.

Our sample is moderately clean. Its purity is given by the lifetime measured on the energy scale^19^ as $k_{\mathrm{B}}^2T_c^2\tau /\hbar E_{{\mathrm{F}}}$, where $k_{\mathrm{B}}$ is the Boltzmann constant and $\hbar$ is the reduced Planck constant. The Fermi energy $E_{\mathrm{F}} = \hbar ^2 k_{\mathrm{F}}^2/2m$ depends on the hole doping. For the hole density of our sample, the Fermi surface is cylindrical rather than spherical, and the Fermi momentum follows from the 2D density of holes in the CuO$_2$ plane $n_{\mathrm{2D}} = n_0c/2 = k_{\mathrm{F}}^2/(2\pi )$, where $c = 11.68$ Å  is the YBaCuO lattice parameter in the *z*-direction. The resulting Fermi energy $E_{\mathrm{F}} = 71$ meV yields the value of $k_{\mathrm{B}}^2T_c^2\tau /\hbar E_{\mathrm{F}} \sim 0.1$, corresponding to the moderately clean sample.

According to Kopnin and Vinokur^19^, the moderately clean d-wave superconductor behaves as the s-wave one. In the absence of reliable formulas for angular frequency $\omega _0$ of quasiparticles bounded in the vortex core in the d-wave superconductors, we used $\hbar \omega _0=(\Delta _0/k_{\mathrm{F}}\xi _0)(1-T^2/T_c^2)$ for the conventional superconductors^26^. We deduced the coherence length $\xi _0$ from the upper critical field in the zero-temperature limit^27^, $B_{c2} = 122~\mathrm{T}=\Phi _0/2\pi \xi _0^2$, where $\Phi _0$ is the magnetic flux quantum. From the energy gap $2\Delta _0 = 4.3 k_{\mathrm{B}}T_c$, we obtained $\omega _0 = 4.4\times 10^{12}$ rad/s, a value close to $4.5\times 10^{12}$ rad/s of our 419 $\mu$m laser line.

To complete the sample characteristics, we assume the pinning of vortices by layer imperfections, for example, the surface roughness. Figure 2e shows the conductivity in the magnetic field of 7 T applied perpendicularly to the film^28,29^. It was interpreted either with the theory specified below or with the model used by Parks^30^, both indicating that the vortex pinning is rather weak with the Labusch coefficient $\kappa \approx 2\times 10^5$N/m$^2$.

### Theoretical prediction

**Figure 3:**
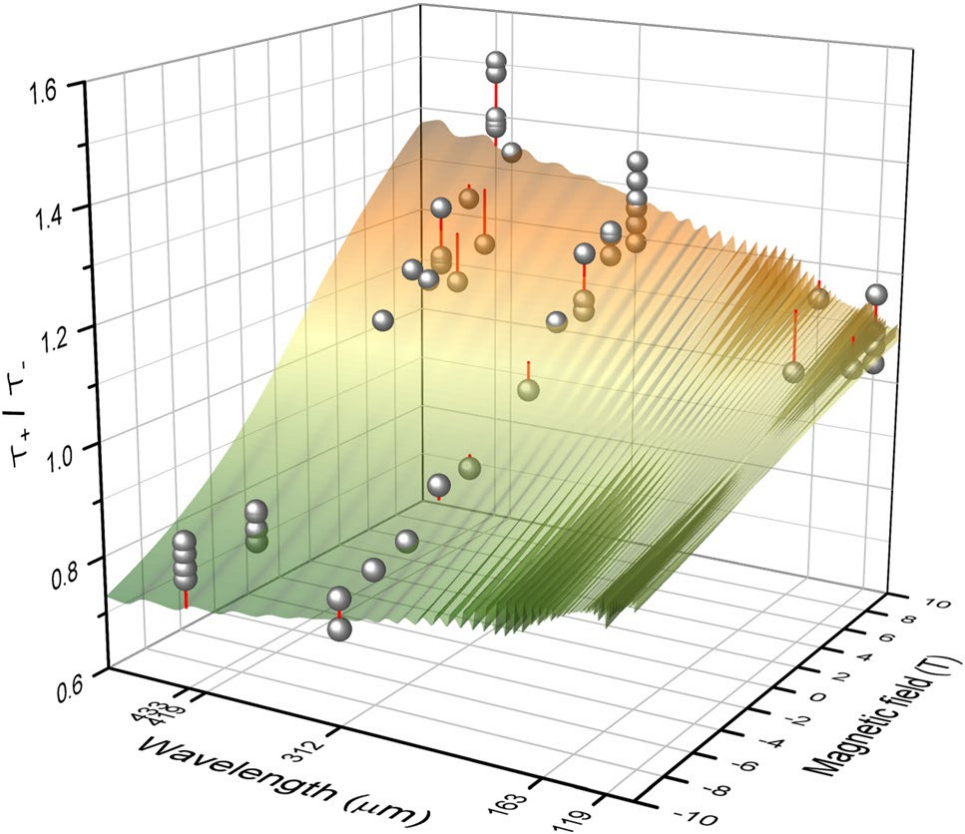
Transmittance ratio ${{\mathcal {T}}}_+/{{\mathcal {T}}}_-$ for the laser lines 119, 163, 312, 419, and 433 $\mu$m as a function of magnetic field. The theoretical prediction (colored surface) with no free fitting parameter is compared with experimental values (spheres) observed at a temperature of 45 K. The corrugation of the theoretical surface results from interference in the film/substrate structure. Its amplitude is smaller than the experimental error. The values observed under identical conditions but in different runs hang on the same vertical line attached to the surface. These repeated measurements demonstrate a spread of the experimental data. Despite the experimental uncertainty, the overall magnitude and dependence on magnetic field and wavelength are consistent with the Kopnin–Vinokur theory.

Our aim is to compare experimental values of ${{{\mathcal {T}}}_+}/{\mathcal{T}_-}$ with a theoretical model. Using the above sample parameters, we can evaluate the film conductivity $\sigma _\pm$ from which the transmittance ${{\mathcal {T}}}_\pm$ results. The theoretical prediction shown in Fig. 3 is based on the Yeh formalism^31^ and covers interferences in the weakly birefringent substrate. For the purpose of discussion, we refer to an approximation ${{{\mathcal {T}}}_+}/{{{\mathcal {T}}}_-} = {|\sigma _-|^2}/{|\sigma _+|^2}$, which differs from the exact theory by less  than 4% as shown in the Supplementary Information^.32^

Kopnin and Vinokur^33^ provide the theoretical conductivities $\sigma _\pm$ derived under very general conditions. Our study allows for two simplifications. First, we focus on temperatures about 45 K, where we observe a large dichroic signal. At such low temperatures, extended quasiparticles are very dilute so we can neglect their contribution to the electric current ${{\varvec{ J}}}$ as well as their effect on vortices. Second, the cyclotron frequency $\omega _c=eB/m$ is small on the scale of the quasiparticle lifetime, $\omega _c\tau \ll 1$, for all experimental magnetic fields, which simplifies dynamics of quasiparticles in the vortex core. Under these conditions, the equation of motion for a fluxon of unitary length takes the form of Newton’s law^33^

1 $$\dot{\varvec p} = \varvec F + \pi \hbar n [ (\varvec J/(en)-\varvec v) \times \varvec z] - \kappa \varvec u$$

with the time derivative of momentum $\varvec{p}$ and the force $\varvec{F}$ from the interaction of the vortex core with the crystal lattice. Unlike Kopnin and Vinokur, we include the pinning force with the Labusch parameter $\kappa$ and vortex displacement $\varvec{u}$ related to its velocity as $\dot{\varvec{u}} = \varvec{v}$. The Magnus force, given by the vector product of the magnetic field direction $\varvec{z} = \varvec{B}/B$ and the vortex velocity related to the condensate current, covers a force by which the flowing condensate acts on the fluxon.

In Kopnin’s model, the fluxon momentum $\varvec{p}$ is a total momentum of quasiparticles in the vortex core which rotate about the vortex axis at an angular frequency $\omega _0$. For our moderately clean sample, the fluxon momentum at the low-temperature limit depends on its velocity as^19^ $\varvec{p} = \mu _\Vert \varvec{v}-\mu _{\perp }[\varvec{v} \times \varvec{z}]$, where

2 $$\begin{aligned} \mu _\Vert =\pi \hbar n \frac{\omega _0\tau ^2}{(1 - i\omega \tau )^2 + \omega _0^2\tau ^2} \, ~~~~~~~ \text {and} ~~~~~~~ \mu _{\perp }=\pi \hbar n\tau \frac{1 - i\omega \tau }{(1 - i\omega \tau )^2 + \omega _0^2\tau ^2} \, . \end{aligned}$$

The diagonal mass $\mu _\Vert$ is complex at finite frequencies, which reflects a delay between a change of the vortex velocity and a change of the total momentum of quasiparticles in its core. The off-diagonal mass $\mu _{\perp }$ describes a property common in anisotropic systems that the velocity of an excitation is not parallel with its momentum.

Quasiparticles disturbed by the vortex motion and the action of FIR light eventually lose their momentum in collisions with impurities and phonons. Via these collisions, the crystal lattice acts on the fluxon by force $\varvec{F}$. With the collision integral approximated by a single relaxation time $\tau$, the force and the momentum are simply related by $\varvec{F}=-\varvec{p}/\tau$. The longitudinal force $-(\mu _\Vert /\tau )\varvec{v}$ is a vortex friction. The transversal force $(\mu _\perp /\tau )[\varvec{v}\times \varvec{z}]$ reduces the Magnus force giving the Kopnin–Kravtsov force^20,21^ in the dc limit.

The electric field in the film^33–35^

3 $$\begin{aligned} \varvec{E} = \frac{1}{\sigma _0} \varvec{J} - [\varvec{v} \times \varvec{B}] \end{aligned}$$

has a skin component $\varvec{J}/\sigma _0$ from the penetrating incident light and an electric field $-[\varvec{v} \times \varvec{B}]$ generated by the vortex motion. At low frequencies, the London conductivity $\sigma _0$ diverges, and the electric field achieves the dc form of the Josephson relation. In the absence of the magnetic field, Eq. (3) reduces to the London formula. Alternatively, one can employ the Coffey-Clem model used by Parks et al.^30^, which is equivalent to Eq. (3).

Conductivities $\sigma _\pm$ are diagonal elements of the conductivity tensor ${\hat{\sigma }} \varvec{E} = \varvec{J}$ in the helical basis. Eigen-vectors of circularly polarized light are constructed from basis vectors in the film plane, e.g., the electric field $\varvec{E} = E_\pm \, \mathrm{e}^{-i \omega t}\varvec{e}_\pm$, where $\varvec{e}_\pm =( {\varvec{x}} \pm i s {\varvec{y}})/\sqrt{2}$ are vectors of the helical basis and $s = \mathrm{sign}(\varvec{B}\cdot \varvec{k})$ reflects the parallel or the anti-parallel orientation of the applied magnetic field $\varvec{B}$ with respect to the wavevector $\varvec{k}$ of incoming light. The eigen-vectors satisfy $[\varvec{e}_\pm \times {\varvec{z}}] = \pm i s\varvec{e}_\pm$; therefore, each of the vector equations (1) and (3) splits into two independent scalar equations for $(+)$ and $(-)$ polarizations. One can easily eliminate the velocity $\varvec{v}$ and write the conductivities

4 $$\begin{aligned} \frac{1}{\sigma _\pm } = \frac{E_\pm }{J_\pm } = \frac{1}{\sigma _0} + \frac{B}{e n} \left( \frac{\omega _0\tau }{1 - i\omega \tau \mp i s {\omega _0\tau }} + i\frac{\kappa }{\pi \hbar \omega n} \right) ^{-1} \end{aligned}$$

needed for the theoretical prediction of the transmittance.

## Discussion

### Experiment versus theoretical prediction

With the full set of sample parameters established from the time-domain spectroscopy, the simplified Kopnin–Vinokur conductivity (4) furnishes us with the theoretical prediction of the circular dichroism. Figure 3 compares this prediction and the experimentally observed dichroism for several values of the applied magnetic field and the THz-laser wavelength. The differences between theory and experiment are smaller than the experimental errors.

With increasing wavelength and field strength, the transmittance ratio ${{\mathcal {T}}}_+/{{\mathcal {T}}}_-$ gradually deviates from unity. The observed trends can be understood in terms of Eq. (3). The field dependence arises from the dominant London contribution $1/\sigma _0$ complemented by the Josephson-type resistivity, which is linear in $\varvec{B}$. The variation with wavelength has a similar cause: for lower frequencies, the London resistivity $1/\sigma _0 \approx -i\omega m /( ne^2)$ is smaller so the Josephson part becomes dominant.

### Vortex mass from experiment

Figure 3 documents that the theory of Kopnin and Vinokur is relevant for the THz dynamics of vortices. Since the observed frequency dependence of magneto-transmission agrees with the theoretical one, even with *no adjustable parameters*, the extrapolation of our results to low frequencies is justified. Based on this, we used their theory to find the vortex mass from our data.

While the sample parameters *n*, $\sigma _0$, and $\tau$ are sound, $\kappa$ and $\omega _0$ are less clear. The Labusch parameter $\kappa$ has a minor effect on the dichroism; therefore, we kept the value $\kappa = 2 \times 10^5$ N/m$^2$. The angular frequency $\omega _0$ was established by the least-square fit of $\mathcal{T}_+/{{\mathcal {T}}}_-$ data. The best-fit value $\omega _0 = 4.3 \times 10^{12}$ rad/s was very close to $4.4\times 10^{12}$ rad/s estimated above. We believe that such close agreement of observed and estimated frequency is fortuitous.

At THz frequencies, where the circular dichroism was found, both diagonal and off-diagonal masses are complex. They become real in the low-frequency limit, as apparent from Eq. (2). Using the experimentally established values of $\omega _0 = 4.3\times 10^{12}$ rad/s and other sample parameters, we evaluated the zero-frequency components of the fluxon mass. In YBa$_2$Cu$_3$O$_{7-\delta }$ at 45 K, the diagonal mass $\mu _\Vert$ amounts to $2.2 \times 10^8~m_e$/cm, while the off-diagonal mass $\mu _\perp$ is more than twice larger, $4.9 \times 10^8~m_e$/cm.

In summary, we have developed a reliable experimental method to measure the circular dichroism of superconducting films threaded by Abrikosov vortices. To interpret our data in terms of vortex dynamics, we have established all the essential material parameters from independent time-domain THz spectroscopy and dc-resistivity measurements. The observed dichroism is in good agreement with the theory of Kopnin and Vinokur based on the circular motion of quasiparticles in the vortex core. Their angular frequency was experimentally determined and used to extrapolate the vortex mass from THz frequencies to low-frequency motion.

## Supplementary Information

Supplementary Information.
