# Supplementary material for: Mass of Abrikosov vortex in high-temperature superconductor YBa2Cu3O7-δ
Source: Sci Rep. 2021 Nov 5;11:21708. doi: 10.1038/s41598-021-00846-x (PMC8571276; doi:10.1038/s41598-021-00846-x)
Supplement: Supplementary file 1 — Supplementary Information. [file 41598_2021_846_MOESM1_ESM.pdf]

Supplementary Information

Mass of Abrikosov vortex in high-temperature superconductor $YBa_2Cu_3O_{7-\delta}$

Roman Tesař, Michal Šindler, Christelle Kadlec, Pavel Lipavský, Ladislav Skrbek, and Jan Koláček

Calculation of transmittance

Our sample comprised a thin YBaCuO film with a thickness of $L = 107$ nm, deposited on a lanthanum aluminate (LAO) substrate. Anisotropic properties of the substrate were measured with standard time-domain THz spectroscopy. For convenience, the x-axis was chosen parallel to the linear polarization of the ordinary ray. At a low temperature of 20 K, we established the substrate thickness $D = 513.5$ μm and dispersion of ordinary and extraordinary refractive indices shown in Figure S1. Measurements at temperatures up to 100 K did not reveal any appreciable deviation from the low-temperature values.

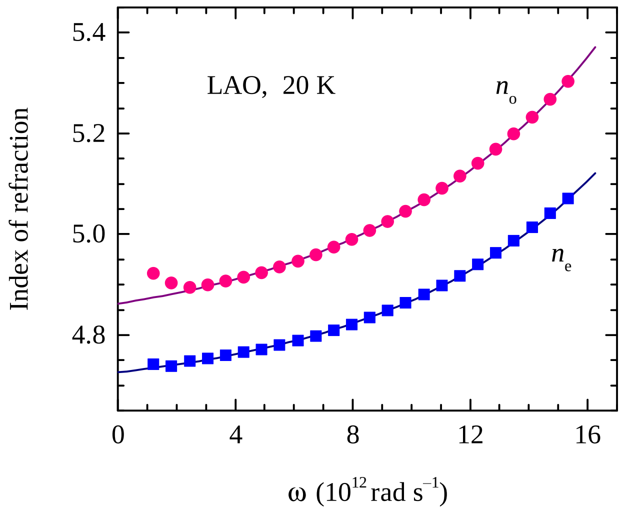

Figure S1. Ordinary (n_o) and extraordinary (n_e) refractive index of lanthanum aluminate (LAO) substrate measured at a temperature of 20 K. The frequency dependence is well described by exponential functions $n_\mathrm{o} = 4.789 + 0.073\exp(0.127\omega)$ and $n_\mathrm{e} = 4.675 + 0.051\exp(0.134\omega)$ with the angular frequency ω in 10^{12} rad/s units (solid lines).

When calculating the sample transmittance, it is necessary to combine two different approaches. Circular dichroism in YBaCuO is naturally described within a vector basis related to the circular polarization of the laser beam. On the other hand, the birefringence of the substrate is conveniently represented in a linear basis associated with the anisotropy axes of LAO. To match these two basis we employ Yeh's 4×4 matrix algebra[1–4].

In each of the segments, vacuum|YBaCuO|LAO|vacuum, we write the resulting electric field $\boldsymbol{E}(z,t) = \boldsymbol{E}(z)\mathrm{e}^{-i\omega t}$ as a sum of four partial waves[1,3]

$$\boldsymbol{E}(z) = \sum_{j=1}^{4} E_j\, \boldsymbol{e}_j\, \mathrm{e}^{ik_j z}\,, \tag{S1}$$

where $\boldsymbol{e}_j$ stand for eigen-polarization vectors and k_j for wavevectors. Partial waves propagating forward are indexed by odd numbers $j = 1, 3$ and waves propagating backward by even numbers $j = 2, 4$. In vacuum and YBaCuO, the eigen-polarization vectors $\boldsymbol{e}_j$ are given in the helical basis as $\boldsymbol{e}_{1,3} = \boldsymbol{e}_{2,4} = (\boldsymbol{x} \pm i\boldsymbol{y})/\sqrt{2}$. In LAO, we use the linear basis $\boldsymbol{e}_{1,2} = \boldsymbol{x}$ and $\boldsymbol{e}_{3,4} = \boldsymbol{y}$. Now we collect the amplitudes E_j of partial waves into a four-component column vector and express the light propagation in a matrix form as

$$\mathbf{E}_{in} = \mathbf{M}\,\mathbf{E}_{tr}\,, \tag{S2}$$

where the 4×4 transfer matrix

$$\mathbf{M} = \mathbf{D}_{\mathrm{vac}}^{-1}\,\mathbf{D}_{\mathrm{film}}\,\mathbf{P}_{\mathrm{film}}\,\mathbf{D}_{\mathrm{film}}^{-1}\,\mathbf{D}_{\mathrm{subs}}\,\mathbf{P}_{\mathrm{subs}}\,\mathbf{D}_{\mathrm{subs}}^{-1}\,\mathbf{D}_{\mathrm{vac}} \tag{S3}$$

connects the incident electric field with the transmitted one via a sequence of dynamical and propagation matrices. The propagation matrices are diagonal. For the YBaCuO film, we have $(\mathbf{P}_{\rm film})_{ij} = \delta_{ij}\mathrm{e}^{ik_jL}$ with $k_{1,2} = \pm n_+\omega/c$ and $k_{3,4} = \pm n_-\omega/c$, where c is the vacuum speed of light and

$$n_\pm = \sqrt{\frac{i\sigma_\pm}{\omega\varepsilon_0}} \tag{S4}$$

is the complex refractive index related to the complex circular conductivity $\sigma_\pm$. Similarly, for the LAO substrate, $(\mathbf{P}_{\rm subs})_{ij} = \delta_{ij}\mathrm{e}^{iq_jD}$ with $q_{1,2} = \pm n_{\rm o}\omega/c$ and $q_{3,4} = \pm n_{\rm e}\omega/c$. Matching of electromagnetic waves at segment interfaces is covered by dynamical matrices

$$\mathbf{D}_{\rm vac} = \frac{1}{\sqrt{2}}\begin{pmatrix} 1 & 1 & 1 & 1 \\ 1 & -1 & 1 & -1 \\ i & i & -i & -i \\ -i & i & i & -i \end{pmatrix}, \qquad \mathbf{D}_{\rm film} = \frac{1}{\sqrt{2}}\begin{pmatrix} 1 & 1 & 1 & 1 \\ n_+ & -n_+ & n_- & -n_- \\ i & i & -i & -i \\ -in_+ & in_+ & in_- & -in_- \end{pmatrix}, \tag{S5}$$

$$\mathbf{D}_{\rm subs} = \begin{pmatrix} 1 & 1 & 0 & 0 \\ n_{\rm o} & -n_{\rm o} & 0 & 0 \\ 0 & 0 & 1 & 1 \\ 0 & 0 & -n_{\rm e} & n_{\rm e} \end{pmatrix}.$$

As already mentioned, we use the same helical basis for vacuum and YBaCuO, which leads to a similarity of $\mathbf{D}_{\rm vac}$ and $\mathbf{D}_{\rm film}$. Since no light approaches the sample from its backside, $(\mathbf{E}_{tr})_2 = 0$ and $(\mathbf{E}_{tr})_4 = 0$, we obtain the transmittances of circularly polarized light in the form

$$\begin{aligned} \mathcal{T}_+ &= \left(|M_{33}|^2 + |M_{31}|^2\right) S, \\ \mathcal{T}_- &= \left(|M_{13}|^2 + |M_{11}|^2\right) S, \end{aligned} \tag{S6}$$

where $S = |M_{11}M_{33} - M_{13}M_{31}|^{-2}$ is a submatrix determinant. The superposition of partial waves in the substrate leads to strong oscillations with the wavelength (see Fig. S2a). These interference effects, however, are considerably reduced in the ratio of transmittances with opposite helicity

$$\frac{\mathcal{T}_+}{\mathcal{T}_-} = \frac{|M_{33}|^2 + |M_{31}|^2}{|M_{13}|^2 + |M_{11}|^2}, \tag{S7}$$

as documented in Fig. S2b.

For purposes of discussion, we can use a free-film approximation which is obtained by sending the substrate thickness to zero, $D \to 0$. A further simplification follows from the expansion in the film thickness according to Hooper and Sambles[5],

$$\frac{1}{\mathcal{T}} = 1 + \frac{\sigma'}{\varepsilon_0\omega}\left(\frac{L\omega}{c}\right) + \frac{|\sigma|^2}{4\varepsilon_0^2\omega^2}\left(\frac{L\omega}{c}\right)^2 + \frac{\sigma'\sigma''}{3\varepsilon_0^2\omega^2}\left(\frac{L\omega}{c}\right)^3 + \frac{1}{12}\left[2\frac{|\sigma|^2}{\varepsilon_0^2\omega^2} + \frac{\sigma''}{\varepsilon_0\omega} + \frac{\sigma''|\sigma|^2}{(\varepsilon_0\omega)^3}\right]\left(\frac{L\omega}{c}\right)^4, \tag{S8}$$

adapted to our notation by substituting for permittivity $\varepsilon = 1 + \mathrm{i}\sigma/(\varepsilon_0\omega)$. This expansion is nearly exact but still too complicated. Numerical calculations reveal that the third term is dominant in our case

$$\mathcal{T} \approx \frac{4\varepsilon_0}{\mu_0 L^2|\sigma|^2}. \tag{S9}$$

We used the approximation (S9) in the main text to discuss the observed dichroism

$$\frac{\mathcal{T}_+}{\mathcal{T}_-} \approx \frac{|\sigma_-|^2}{|\sigma_+|^2}. \tag{S10}$$

Its validity is demonstrated in Fig. S2b.

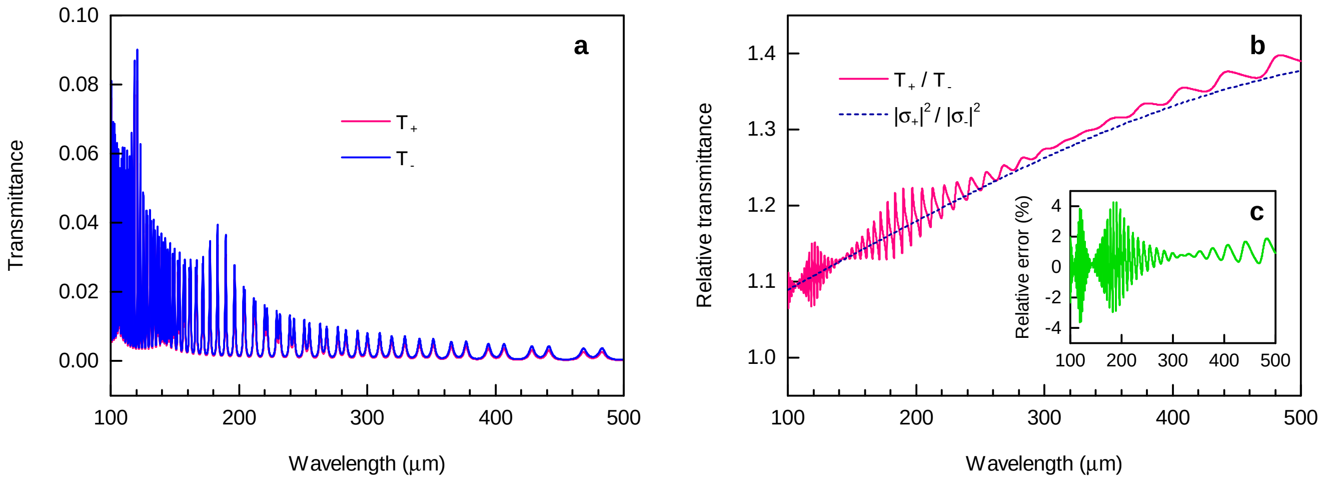

Figure S2. Transmittance as a function of the laser wavelength for $B = 10$ T and $T = 45$ K. **(a)** Interference in the sample substrate leads to strong oscillation in the transmittance. Due to weak birefringence of the substrate, both circular polarizations follow similar but not identical pattern. **(b)** The interference almost cancels in the ratio of transmittances (red line). The free-film approximation (blue line) differs from the exact result by less than 4%, as detailed in the inset **(c)**.

References

1. Yeh, P., Electromagnetic propagation in birefringent layered media, J. Opt. Soc. Am. **69**, 742 (1979).
2. Yeh, P., Optics of anisotropic layered media: A new 4×4 matrix algebra, Surf. Sci. **96**, 41 (1980).
3. Višňovský, Š., *Optics in Magnetic Multilayers and Nanostructures* (CRC Press, 2006).
4. Šindler, M., Tesař, R. , Koláček, J., and Skrbek, L., Interpretation of transmission through type-II superconducting thin film on dielectric substrate as observed by laser thermal spectroscopy, Physica C **483**, 127 (2012).
5. I. R. Hooper, and J. R. Sambles, Some considerations on the transmissivity of thin metal films, Opt. Express **16**, 17258 (2008).
